# Repurposing as a strategy for the discovery of new anti-leishmanials: the-state-of-the-art

**DOI:** 10.1017/S0031182017000993

**Published:** 2017-08-14

**Authors:** REBECCA L. CHARLTON, BARTIRA ROSSI-BERGMANN, PAUL W. DENNY, PATRICK G. STEEL

**Affiliations:** 1 Department of Chemistry, University Science Laboratories, South Road, Durham DH1 3LE, UK; 2 Instituto de Biofísica Carlos Chagas Filho, Universidade Federal do Rio de Janeiro, Ilha do Fundão, CEP 21·949-900 Rio de Janeiro, RJ, Brazil; 3 Department of Biosciences, University Science Laboratories, South Road, Durham DH1 3LE, UK

**Keywords:** leishmaniasis, repurposing, repositioning, drug discovery

## Abstract

Leishmaniasis is a vector-borne neglected tropical disease caused by protozoan parasites of the genus *Leishmania* for which there is a paucity of effective viable non-toxic drugs. There are 1·3 million new cases each year causing considerable socio-economic hardship, best measured in 2·4 million disability adjusted life years, with greatest impact on the poorest communities, which means that desperately needed new antileishmanial treatments have to be both affordable and accessible. Established medicines with cheaper and faster development times may hold the cure for this neglected tropical disease. This concept of using old drugs for new diseases may not be novel but, with the ambitious target of controlling or eradicating tropical diseases by 2020, this strategy is still an important one. In this review, we will explore the current state-of-the-art of drug repurposing strategies in the search for new treatments for leishmaniasis.

## INTRODUCTION: DRUG REPOSITIONING

**Fig. 1.**
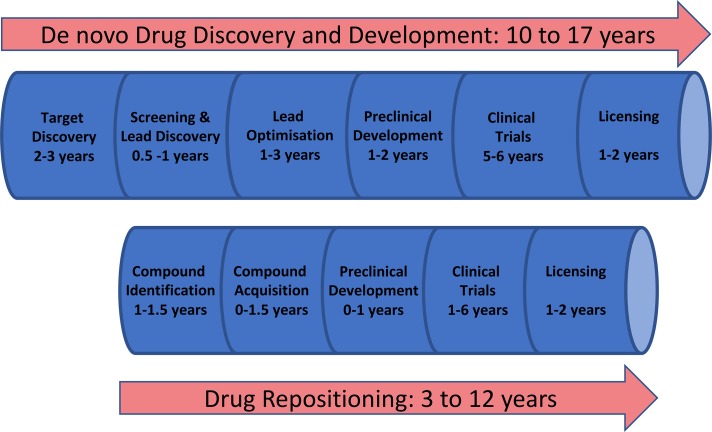
Drug discovery and repositioning pathways (adapted from Guha *et al*. 2015).

The introduction of a new drug from initial concept to public release is a slow expensive process. Developing a new chemical entity (NCE) drug and delivering it to market, *de novo* drug discovery (Fig. 1), is estimated to take 10–17 years and cost ~$1·8 billion (Paul *et al.* 2010). Moreover, the probability of success is lower than 10% and the costs of late stage failures in this process are significant and are accounted for in the high cost of patented drugs. Consequently, the discovery of new drugs for leishmaniasis, and other neglected tropical diseases, for which the average patient suffering from visceral leishmaniasis (VL) exists on an income of less than $2 per day (WHO, 2002), is simply not a commercially viable operation for pharmaceutical companies without significant cross-subsidy.

**Fig. 2.**
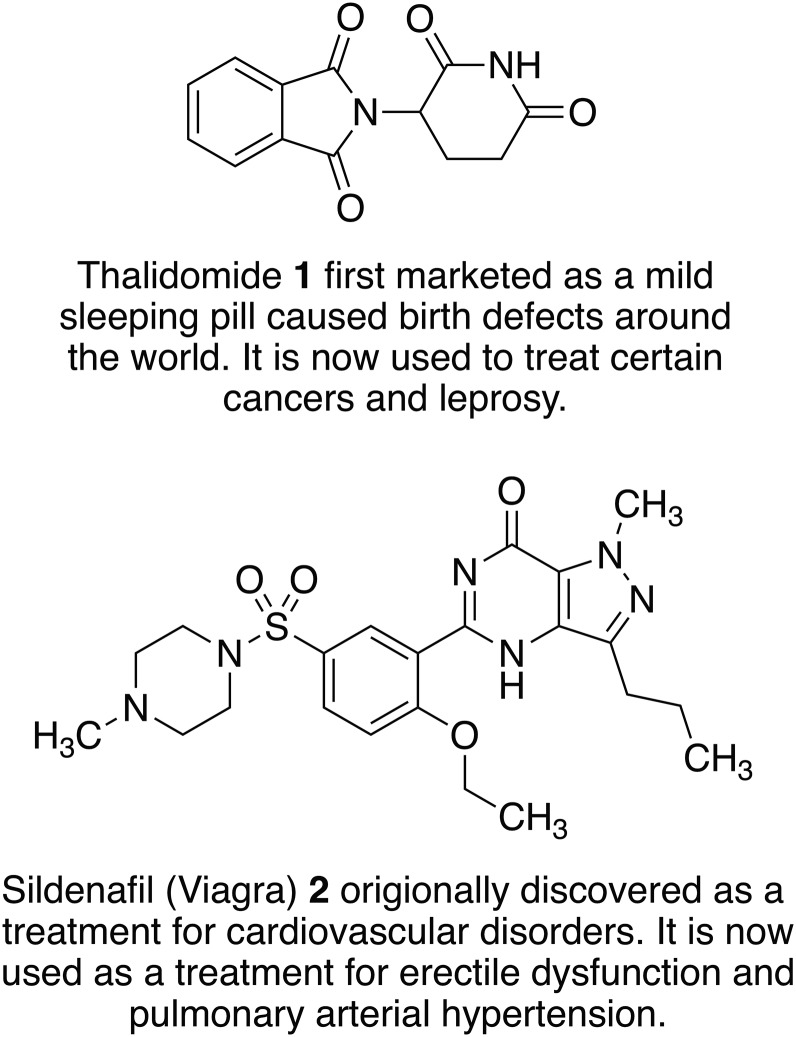
Examples of old drugs directly repositioned as treatments for new diseases and dysfunctions.

Alternative strategies are therefore needed and the process of drug repositioning or repurposing, where new applications for existing drugs or drug candidates are discovered and refined, has become increasingly common (Ashburn and Thor, 2004; Aubé, 2012; Oprea and Mestres, 2012; Andrews *et al.* 2014; Klug *et al.* 2016; Jain and Sharma, 2017). When using a drug repositioning strategy several development phases necessary to develop NCE drugs can be bypassed or considerably shortened (Fig. 1) because candidates will have passed various checkpoints and information is available about their pharmacokinetic and safety profiles. Reflecting this, the process of finding new uses for drugs outside their original indication is becoming increasingly successful as more companies are screening their libraries for repositioning candidates. Figure 2 illustrates two examples of approved drugs possessing optimum properties for their new indication. However, it should be noted that these cases are relatively rare and some development work is still commonly required. Moreover, whilst serendipity (phenotypic screening) can play a sizeable role in identifying repurposed drugs, it is important to establish the molecular connection between old drugs and new targets as this information aids the development of the new therapeutics. Despite these caveats drug repositioning can reduce time, cost and risk, and is a particularly attractive approach for neglected tropical diseases where new medications are needed urgently to treat the poorest of people. In this review, we will focus on the neglected tropical disease, leishmaniasis, and how repositioning strategies have shown potential to enable the discovery of new medicines for this disease.

## LEISHMANIASIS

Leishmaniasis is an important vector borne ´Neglected Tropical Disease’ caused by protozoan parasites of the genus *Leishmania* (Roberts, 2006). With over 350 million people world wide considered at risk, 12 million people currently infected (Kedzierski, 2010), one million new cases reported annually, more than 30 000 deaths each year (WHO, 2016) and an economic cost that can be best estimated in terms of 2·4 million disability adjusted life years, the health challenge is only surpassed amongst parasitic diseases by malaria, schistosomiasis and lymphatic filariasis (Davies *et al.* 2003; Bern *et al.* 2008). Consequently, leishmaniasis has been classified by the World Health Organization as Category I: emerging or uncontrolled diseases. In particular, the spread and severity of infection is exacerbated by its status as an important co-infection of AIDS patients and the overlap in prevalence of HIV and *Leishmania* spp. (Alvar *et al.* 2008). The disease is endemic in 98 countries across five continents and has strong links with poverty, principally affecting low income regions of Africa, Asia and Latin America (Alvar *et al.* 2012). However, the impact of the disease is spreading and, reflecting population movements and climate change, leishmaniasis has also been recorded with growing frequency in Southern Europe (Gradoni, 2013).

Leishmaniasis is transmitted through the bite of infected female phlebotomine sandflies. Subsequently, in the mammalian host, flagellated promastigote *Leishmania* spp. are taken up by macrophages where they transform into the non-flagellated amastigote form, proliferate and cause disease. Infected macrophages can then be taken up by the insect vector during a second blood meal, the parasite then transforms back to the promastigote form and the cycle is perpetuated. Importantly, many *Leishmania* spp. can infect multiple mammalian species leading to animal reservoirs for human infection, most notably in canines (Solano-Gallego *et al.* 2011).

Human disease is caused by 20 of the 31 parasite species that infect mammals with the different species leading to different clinical manifestations (Akhoundi *et al.* 2016). Depending on parasite tropism for skin or visceral macrophages, and host immune status, clinical forms range from localized cutaneous leishmaniasis (CL), diffuse CL, disseminated CL, mucosal CL, to visceral leishmaniasis (VL). The most common form of the disease is CL which, although not fatal, leads to slow growing skin lesions, which leave permanent scars and in some cases cause serious disability. Mucocutaneous leishmaniasis is the most disfiguring form and causes destruction of soft tissue in the nose, mouth and throat. In contrast, VL is the most severe form of the disease as it affects the vital organs of the body and is fatal if left untreated (CDC, 2016).

Treatment of leishmaniasis is challenging and there are currently no vaccines or prophylactic drugs to prevent human infection. For over 70 years, the mainstay of antileishmanial therapy has been pentavalent antimonials (Pentostam **3** and Glucantime **4**) which are administered by slow intravenous or intramuscular injections (Kedzierski *et al.* 2009). The mode of action is not fully understood but one possibility involves the biological reduction of Sb(V) to Sb(III) by the parasite or by infected host cells to create antileishmanial activity (Shaked-Mishan *et al.* 2001; Krauth-Siegel & Comini 2008). Unfortunately, antimonials are also toxic (Sundar and Chakravarty, 2010*b*) and reports of resistance are becoming increasingly common. For example, a study of the efficacy of Pentostam **1**, in North Bihar, India, revealed that only 36% of patients could be cured when treated with a dose of 20 mg kg^−1^ for 30 days (Croft *et al.* 2006).

For the past seven decades, amphotericin B **5** (AmpB) and pentamidine **6** have been used in cases of antimonial failure (Kedzierski *et al.* 2009). Pentamidine **6** is an antimicrobial medication that was commonly used as a second line, parenteral, treatment for leishmaniasis in the case of antimonial failure. The primary mode of action of pentamidine **6** in kinetoplastids is not well understood but there is some evidence that it inhibits the active transport system and disrupts the mitochondrial membrane potential. However, pentamidine **6** has lower efficacy than amphotericin B **5** (Lockwood and Moore, 2010) and this, coupled with severe toxicity, have resulted in a gradual decline in its use (No, 2016).

The antifungal drug AmpB **5**, first isolated in 1955, was reported to have antileishmanial activity in the early 1960s. AmpB **5** binds to ergosterol, forming pores in the membrane, which leads to the death of the parasite (Kamiński, 2014). Whilst severe side effects limited its use, liposomal formulations that circumvent these problems have been developed. However, a course of L-AmpB **5** still requires parenteral administration and, without extensive subsidy is expensive (up to $3000 per course). This makes L-AmpB inaccessible to most patients who live in the poorest, least developed regions. In comparison, an equivalent course of pentavalent antimonials costs between $150 and $198 (de Menezes *et al.* 2015). Despite this, L-AmpB **5** is now often preferred over Penstostam **3** for VL because it is less toxic and there are fewer reports of parasitic resistance (Croft *et al.* 2006; Sundar and Singh, 2016).

Along with L-AmpB, paromomycin **7** and miltefosine **8** are now approved drugs for VL (Sundar and Chakravarty, 2015). Paromomycin **7** is an antibiotic and a relatively new therapy for the parenteral treatment of VL in India. It has been shown to be effective and relatively cheap first-line treatment with low toxicity, requiring a course of 15 mg kg^−1^ for 21 days (Musa *et al.* 2010; No, 2016). The mechanism of action is not fully understood but it is thought to inhibit protein synthesis (Fernández *et al.* 2011). As with the other treatments there are drawbacks, the drug is administered by injection (Sundar *et al.* 2007) and shows poor efficacy in some regions in Africa (Hailu *et al.* 2010).

As discussed above, these current medications all involve prolonged parenteral administration, which leads to many complications, including poor patient compliance, blood-borne disease from unsanitary conditions and the need for medical facilities. Therefore, in the past two decades there has been a focus on developing new oral therapies. In 2002 miltefosine **8**, originally developed as an anticancer agent, was introduced as the first oral antileishmanial agent (Soto and Berman, 2006). The primary mechanism of action is poorly understood, although the drug is thought to involve disruption of ether-phospholipid metabolism, glycosylphosphatidylinositol anchor biosynthesis and signal transduction within the parasite (No, 2016). Miltefosine **8** efficacy against both VL and CL has been reported and, in 2014, the US Food and Drug Administration approved the drug for all forms of leishmaniasis (Dorlo *et al.* 2012). However, this oral therapy does have limitations, including high costs and teratogenic effects. Moreover, the long-term effectiveness of miltefosine **8** is questionable, with reports of VL relapses in Nepalese patients treated with this drug. In addition, miltefosine **8** resistance is readily induced in *Leishmania donovani in vitro* (Seifert *et al.* 2003).

**Fig. 3.**
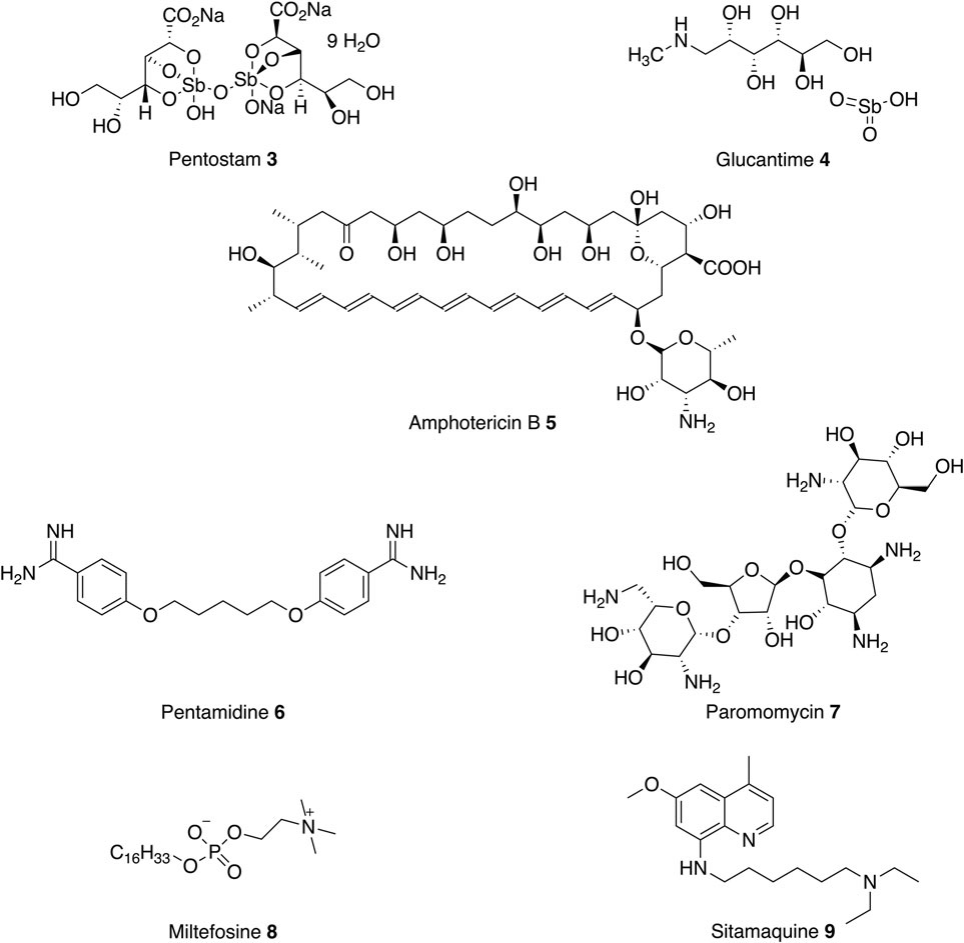
Current antileishmanials.

In conclusion, all current treatments (Fig. 3) have serious limitations such as high cost, route of administration, severe toxic side effects and increasing drug resistance. Currently, there are some new therapies under clinical investigation as monotherapies or in combination with existing antileishmanials (Bahamdan *et al.* 1997; Alrajhi *et al.* 2002; Momeni *et al.* 2002; Machado *et al.* 2007; Sundar *et al.* 2011; ClinicalTrials.gov, 2016). These include paromomycin **7** topical cream, currently in phase 3 trials as a treatment for CL, and sitamaquine **9** in phase 2 to treat VL.

## REPOSITIONING OF DRUGS TARGETING LEISHMANIASIS

Significantly, as noted above in the Introduction, many of the existing therapies for leishmaniasis are licensed or were initially intended for other illnesses. For example, Amphotericin B **5** is an antifungal drug, paromomycin **7** is an aminoglycoside antibiotic used to treat intestinal infections, and miltefosine **8** was originally developed as an anticancer agent. This section will review drugs for different disease states; cancer, microbial infection, depression, allergies and others; that have shown potential to be repurposed as new antileishmanial therapies, and discuss their putative parasitic drug targets.

It should be noted that many natural products often used in traditional medicines have antileishmanial activity, including vast numbers of different alkaloids (Mishra *et al.* 2009*a*, *b*), flavonoids (Mittra *et al.* 2000; da Silva *et al.* 2012) chalcones (Chen *et al.* 1994; Boeck *et al.* 2006; Aponte *et al.* 2010; de Mello *et al.* 2014) and terpenoids (Rosa *et al.* 2003; Torres-Santos *et al.* 2004; Arruda *et al.* 2005; Mazoir *et al.* 2011). These compounds have been discussed extensively in previous literature (Mishra *et al.* 2009*a*, *b*; Schmidt *et al.* 2012; Singh *et al.* 2014*a*) however, they are structurally complex molecules, often affecting multiple targets and possess properties that make them unlikely to be orally active drugs. As such the greatest potential of natural products is for the identification of novel targets required to underpin target-based drug discovery and not as starting points for a repositioning strategy and therefore they will not be discussed further in this review.

### Anticancer drugs

**Fig. 4.**
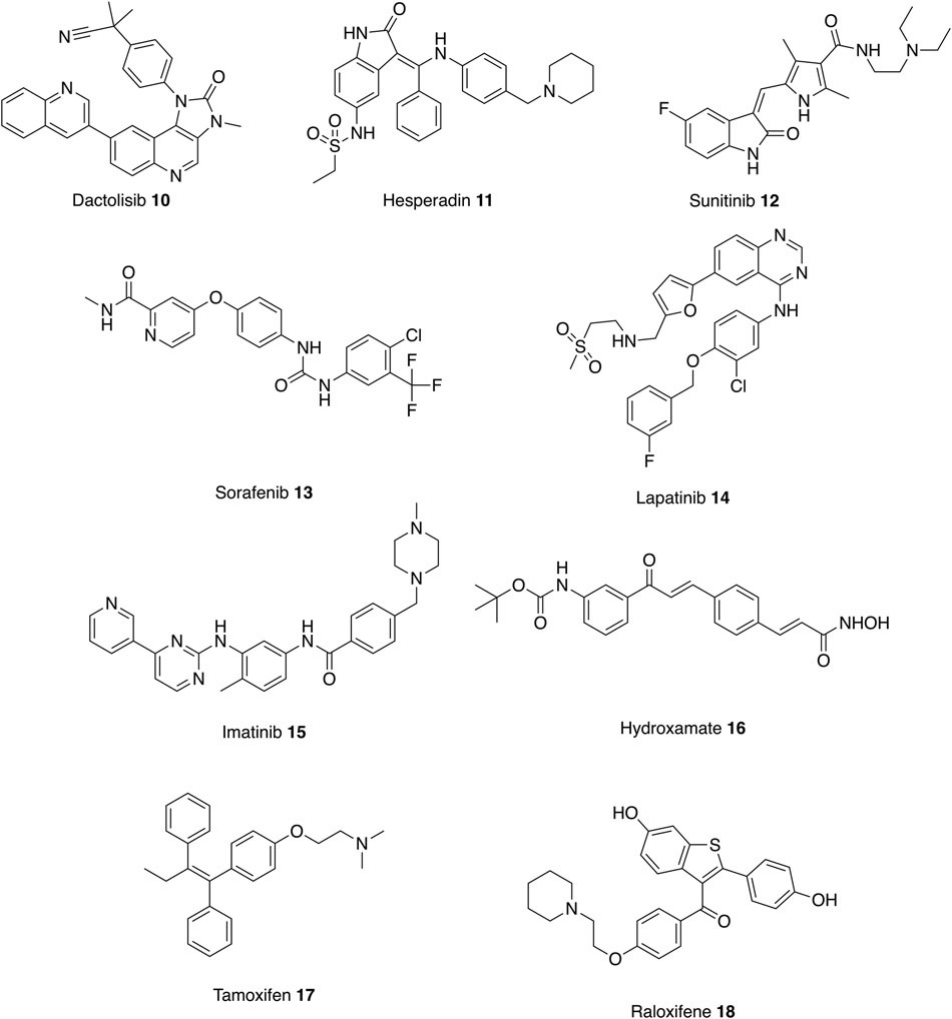
Treatments for cancer that could be repurposed as antileishmanials.

Both cancerous cells and *Leishmania* spp. have the ability to proliferate in a host organism for prolonged periods of time and some enzymes targeted by anticancer therapies can also be used in the development of antileishmanials (Fig. 4, Table 1) (Klinkert and Heussler, 2006; Uliana and Barcinski, 2009).

**Table 1.** List of drugs in this review and their activities

Drug	Species	*In vitro* form	EC_50_ (*μ*m)	Selectivity index	Animal study	References
10	*L. major*	Promastigotes	0·11	–	Y	Diaz-Gonzalez *et al.* (2011)
	*L. donovani*	Promastigotes	0·14		N	
		Axenic amastigotes	0·07			
11	*L. major*	Promastigotes	0·12	0·08	N	Patel *et al.* (2014)
		Axenic amastigotes	2·37			
12	*L. donovani*	Intracellular amastigotes	1·08	7·04	Y	Sanderson *et al.* (2014)
	*L. mexicana*	Intracellular amastigotes	2·63	2·89	N	
13	*L. donovani*	Intracellular amastigotes	3·73	1·88	Y	
	*L. amazonensis*	Intracellular amastigotes	6·87	1·02	N	
	*L. mexicana*	Intracellular amastigotes	4·72	1·48	N	
	*L. major*	Intracellular amastigotes	3·77	1·86	N	
14	*L. donovani*	Intracellular amastigotes	2·49	2·41	Y	
15	*L. amazonensis*	–	–	–	Y	Wetzel *et al.* (2012)
16	*L. donovani*	Axenic amastigotes	0·47	10	N	Wang *et al.* (2015)
17	*L. amazonensis*	Promastigotes	13·51	–	Y	Trinconi *et al.* (2016)
		Intracellular amastigotes	4·25			
18	*L. amazonensis*	Promastigotes	30·2	1·77	Y	Reimao *et al.* (2014)
		Intracellular amastigotes	16·2			
19	*L. major*	Intracellular amastigotes	>105	–	Y	Berman and Gallalee (1987)
20	*L. major*	–	–	–	Y	Zakai and Zimmo (2000)
21	*L. major*	Promastigotes	136	1·82	Y	Shokri *et al.* (2017)
		Intracellular amastigotes	24			
22	*L. amazonensis*	Promastigotes	0·44	187·5	Y	de Macedo-Silva *et al.* (2013), Zakai and Zimmo (2000)
		Intracellular amastigotes	0·08			
23	*L. amazonensis*	Promastigotes	2·74	12·2	Y	de Macedo-Silva *et al.* (2013)
		Intracellular amastigotes	1·63			
24	*L. infantum*	Promastigotes	2–8	3·6	N	Mesquita *et al.* (2014)
		Intracellular amastigotes	11			
25	*L. major*	Intracellular amastigotes	0·015	>407	N	Khraiwesh *et al.* (2016)
26			0·061	>100		
27			0·091	14·2		
28			0·13	>45·4		
29	*L. infantum*	*Promastigotes*	139	3·09	N	Mesquita *et al.* (2013)
		*Intracellular amastigotes*	22			
	*L. donovani*	*–*	–	–	Y	Zhang *et al.* (2010)
	*L. amazonensis*	*Promastigote*	7·2	115	N	Nava-Zuazo *et al.* (2014)
30	*L. donovani*	Promastigotes	5·6	>17·9	Y	Wyllie *et al.* (2012)
		Axenic amastigotes	2·8			
		Intracellular amastigotes	Inactive	–		
31	*L. amazonensis*	–	–	–	Y	Rodrigues *et al.* (2006)
32	*L. amazonensis*	–	–	–	Y	Rodrigues *et al.* (2006)
33	*L. donovani*	Promastigotes	0·9	>10	Y	Patterson *et al.* (2013)
		Intracellular amastigotes	4·92			
34	*L. donovani*	Promastigotes	0·16	>54	Y	Patterson *et al.* (2013)
		Intracellular amastigotes	0·93			
35	*L. donovani*	Promastigotes	0·016	>3125	Y	Patterson *et al.* (2016)
		Axenic amastigotes	0·005	>10 000		
		Intracellular amastigotes	0·087	>575		
36	*L. donovani*	Intracellular amastigotes	Inactive	–	Y	Smith *et al.* (2000)
37	*L. donovani*	Promastigotes	Inactive	–	N	Buates and Matlashewski (1999)
		Axenic amastigotes	Inactive	–		
		Intracellular amastigotes	Active	–		
	*L. major*	Intracellular amastigotes	21–42	–	Y	El-On *et al.* (2007)
38	*L. infantum*	Promastigotes	34	4·14	Y	Pinto *et al.* (2014)
		Intracellular amastigotes	21			
39	*L. donovani*	Intracellular amastigotes	Active	–	N	Clark *et al.* (2007)
40	*L. mexicana*	Promastigotes	1·24	–	N	Navin *et al.* (2017)
41	*L. donovani*	Intracellular amastigotes	4·90	–	Y	Chan *et al.* (1998)
42	*L. donovani*	Intracellular amastigotes	8·60	0·55	N	Parveen *et al.* (2005)
43	*L. donovani*	Promastigotes	>50	9·68	N	De Muylder *et al.* (2011)
		Axenic amastigotes	>50			
		Intracellular amastigotes	3·45			
44	*L. donovani*	Promastigotes	17·30	3·02	N	De Muylder *et al.* (2011)
		Axenic amastigotes	>50			
		Intracellular amastigotes	5·40			
45	*L. donovani*	Promastigotes	37	3·51	N	Singh *et al.* (2014*b*)
	*L. donovani*	Intracellular amastigotes	28			
46	*L. donovani*	Promastigotes	21	2·16	N	Dinesh *et al.* (2014)
	*L. donovani*	Intracellular amastigotes	46			
47	*L. donovani*	Promastigotes	23·8	>13	N	Dinesh *et al.* (2015)
		Intracellular amastigotes	7·5			
48	*L. mexicana*	Promastigotes	0·9	–	Y	Serrano-Martin *et al.* (2009*b*)
		Intracellular amastigotes	0·008			
49	*L. mexicana*	Promastigotes	0·115	–	N	Benaim *et al.* (2014)
		Intracellular amastigotes	0·0007			
50	*L. major*	Promastigotes	0·41	1	N	Firdessa *et al.* (2015)
		Intracellular amastigotes	4			
51	*L. donovani*	Promastigotes	0·25	–	N	Witschel *et al.* (2012)

Kinases have long been recognized as a major target in oncology and this has spawned a number of efforts to repurpose a range of kinase inhibitors for leishmaniasis. For example, miltefosine **8** is the first, and still the only, oral drug in the treatment of VL but was originally developed as a treatment for breast cancer. Like many anticancer drugs, miltefosine **8** induces apoptosis through kinase inhibition. In this case the PI3K/Akt/PkB pathway is targeted and it has been reported that inhibition of this pathway may influence the susceptibility of host cell to *Leishmania* infection (Dorlo *et al.* 2012).

The genomes of trypanosomatids encode at least 12 proteins belonging to the PI3K protein superfamily, many of which are unique to the parasites. The Target of Rapamycin (TOR) kinase is a member of the PI3K-related kinase (PKK) subfamily and has a central role in fundamental processes such as growth, cell shape and autophagy. Trypanosomatids possess four distinct genes belonging to the TOR family whereas mammals only possess one mTOR protein. On this basis, a targeted set of eight known mTOR inhibitors with varied potencies and selectivity for mTOR/PI3K were screened against *Trypanosoma brucei*, *Trypanosoma cruzi*, cutaneous *Leishmania major* and visceral *L. donovani.* From these data, the mTor/PI3K inhibitor, Dactolisib **10**, was identified as being the most active against all the species tested and therefore was tested in animal models of *T. brucei rhodesiense*, *T. cruzi* and *L. major*. Unfortunately, no efficacy was observed against either *T. cruzi* or *L. major*; this may reflect the need for the drug to cross into the phagolysosomal compartment where the parasite resides. In contrast, and consistent with this hypothesis, Dactolisib significantly reduced the parasite burden and extended the survival of mice infected with *T. brucei rhodesiense* (Diaz-Gonzalez *et al.* 2011), a blood stream parasite. Although the exact mechanism of action has yet to be identified this investigation has demonstrated a potential target in the search for antitrypanosomal therapies.

Similar small scale focused screens against specific kinase targets have been conducted. For example, through exploring Aurora kinases, hesperidin **11** has been identified as a putative antileishmanial lead but host cell toxicity was observed when the compound was screened against the hepatic cancer cell line (Patel *et al.* 2014). Although, further structure-activity relationship (SAR) studies demonstrated that these inhibitors are not general toxins but can be modified to improve selectivity, useful antileishmanial selectivity was not obtained. More significantly, and consistent with other reports (De Muylder *et al.* 2011), there was no correlation between activity against promastigote and axenic amastigote with in this case the latter being the less sensitive form.

Whilst target-based screening is an attractive strategy, the need for a specific orthologous target protein may not be essential as many kinase inhibitors affect multiple proteins and simply the proven cellular accessibility of these compounds is a sufficient starting point. For example, whilst tyrosine kinases are not expressed in trypanosomatids, protein phosphorylation occurs in parasites and therefore kinase inhibitors may interact with other enzymes (Karaman *et al.* 2008; Peña *et al.* 2015). Reflecting this, in a screen of eleven tyrosine kinase inhibitors, sunitib **12**, sorafenib **13** and lapatinib **14** showed antileishmanial activity against intracellular *L. donovani* amastigotes (Sanderson *et al.* 2014). Control experiments confirmed that the leishmanial targets were unlikely to be the same as those found in mammalian cells and these kinase inhibitors showed a degree of selectivity against amastigotes compared with mammalian cells. In addition, an oral dose of sunitib **12**, sorafenib **13** and lapatinib **14** reduced liver parasite burden in mice infected with *L. donovani*. Whilst these screens identify new leads, the challenge for such studies is in the identification of the actual target to guide further optimization.

Kinases also proved to be the most common predicted target from a set of 192 antileishmanial compounds identified from a high throughput phenotypic screen of 1·8 million compounds against representative kinetoplastid species, including *L. donovani* (Peña *et al.* 2015). However, since most of these compounds have no literature precedent this was not strictly a repurposing study, and further discussion is outside the scope of this review. Finally, an interesting, and somewhat different, study involving the repurposing of kinase inhibitors, was the use of imatinib **15** (Gleevec, another tyrosine kinase inhibitor used in the treatment of multiple cancers) to target the host macrophage AbI and Arg kinases believed to be involved in parasite entry to the cell. Mice infected with *L. amazonenesis* were treated with oral imatinib, which resulted in smaller lesions that developed later and a reduction in parasite burden relative to controls. (Wetzel *et al.* 2012). Imatinib is a clinically approved drug with relatively benign side effects when compared with antileishmanials on the market. It would be of great benefit to further understand the cell entry pathway to provide new lines of therapy for leishmaniasis.

The approach of starting with inhibitors with known modes of action, for example against mammalian kinases, and screening for new antileishmanial activities for which orthologous protozoal targets can then be identified is an attractive concept. However, good levels of host parasite selectivity are required before any hits can be repurposed as antiparasitic therapeutics. This was nicely illustrated in a study exploring Lysine Deacetylases (KDAC) as putative antileishmanial drug targets. KDACs are one of the most studied epigenetic drug targets of humans (Wang *et al.* 2015) and inhibition of their activity is a validated strategy for cancer treatment (Patil *et al.* 2010). As with the kinases discussed above, *Leishmania* spp. have multiple genes encoding different HDACs isoenzymes some of which are essential to parasite survival (Patil *et al.* 2010). Whilst, the homology between kinetoplastid and mammalian KDACs is low (~40%) much higher levels of similarity are found in active site residues (Wang *et al.* 2015). Reflecting this only one compound, Hydroxamate **16**, showed significant selectivity (~10-fold) between host cell and parasite and high (nanomolar) activity against axenic *L. donovani* amastigotes (Wang *et al.* 2015).

A final class of anticancer agents that have been shown to have potential as antileishmanials are the selective estrogen-receptor modulators, tamoxifen **17** and raloxifene **18**, used to prevent and treat breast cancer. Both have been identified as potential candidates for leishmaniasis treatment with micro-molar potency against intracellular *Leishmania amazonensis* amastigotes (Reimao *et al.* 2014; Doroodgar *et al.* 2016; Trinconi *et al.* 2016). The mechanism of action of tamoxifen **17** and raloxifene **18** is still unclear as estrogen responses have not been described in *Leishmania* spp. (Miguel *et al.* 2007, 2008). However, it has been postulated that tamoxifen **17** causes changes in parasite-membrane properties and disrupts sphingolipid synthesis (Trinconi *et al.* 2016), whereas raloxifene **18** is thought to damage the cell membrane and the mitochondrion of the parasite (Reimao *et al.* 2014). Whilst the antileishmanial activity of these compounds has not necessarily been optimized, the proven safety profile of these repurposed drugs allows them to be considered as synergists in combination therapies to help combat growing concerns about the emergence of parasitic resistance. For instance, combination of tamoxifen **17** and miltefosine **8** *in vitro* and *in vivo* revealed no interaction between the two drugs and suggested that tamoxifen could slow the emergence of miltefosine **8** resistance (Trinconi *et al.* 2016).

### Antimicrobial drugs

**Fig. 5.**
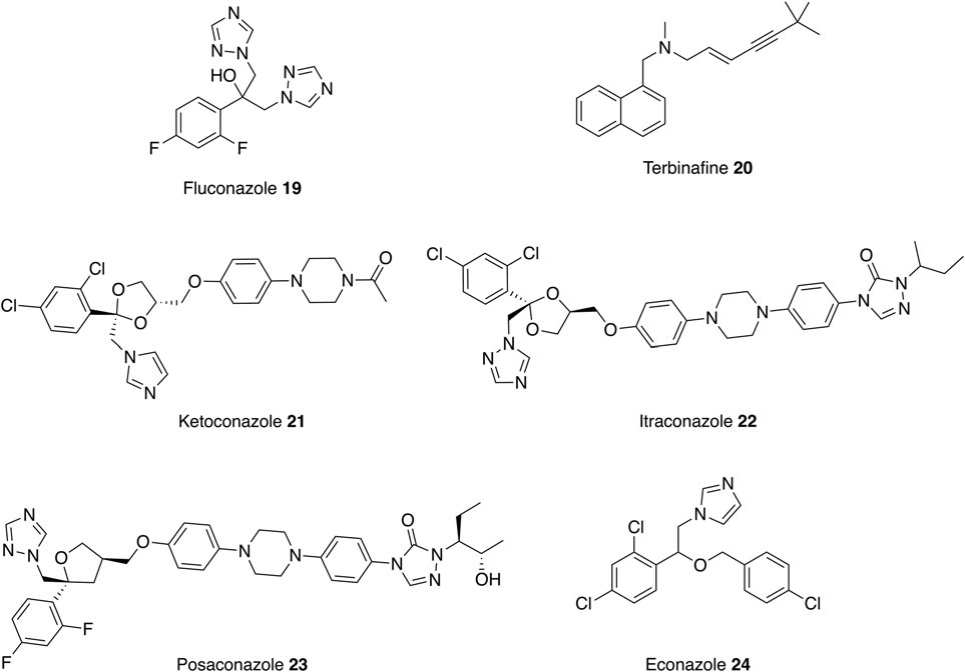
Antifungal drugs that could be repurposed as antileishmanials.

**Fig. 6.**
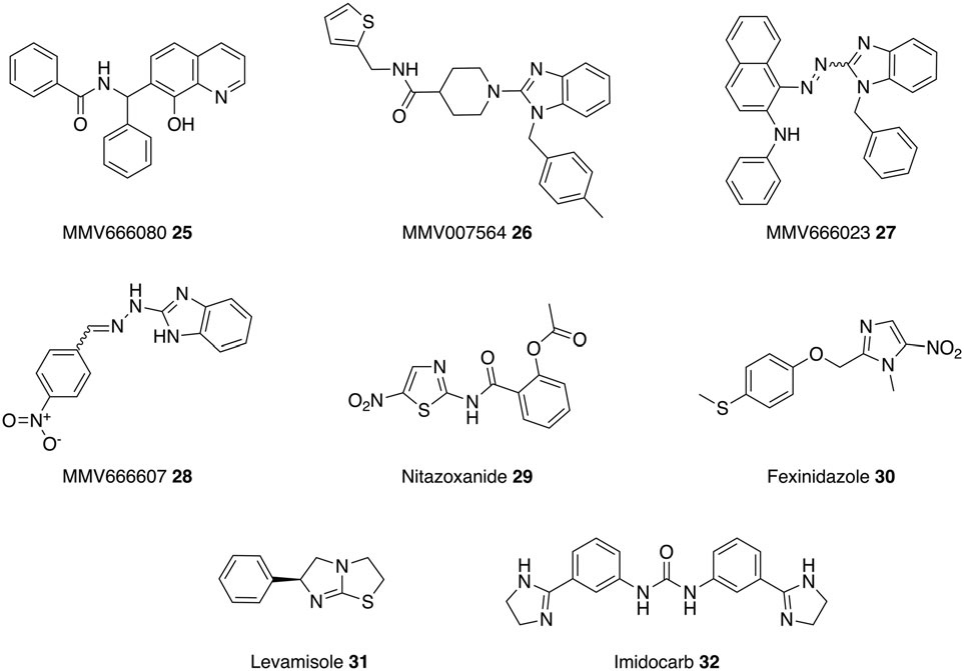
Antiparasitic drugs that could be repurposed as antileishmanials.

**Fig. 7.**
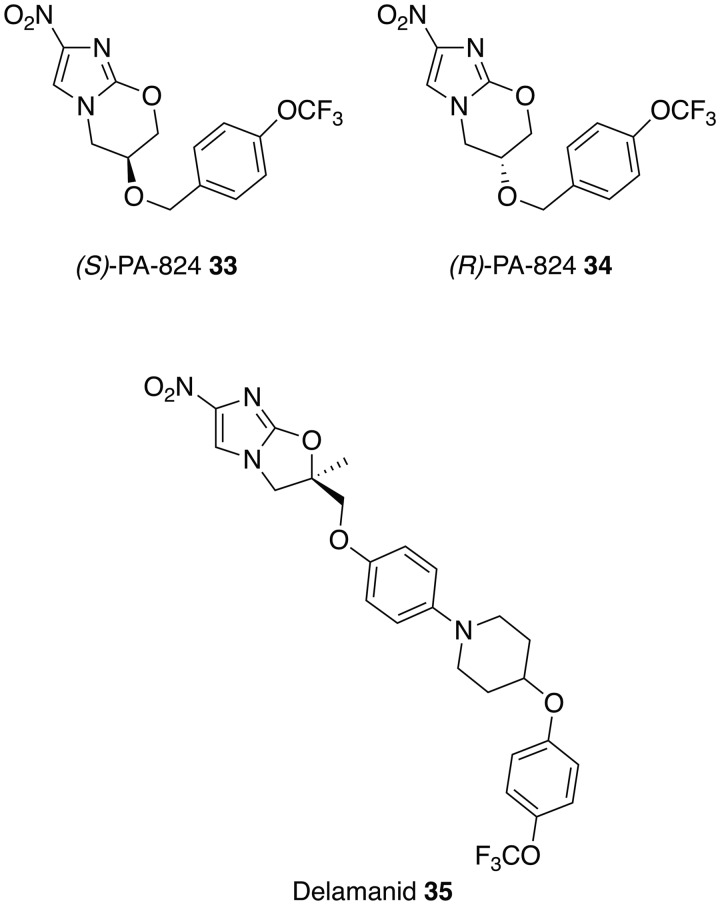
Antibacterial drugs that could be repurposed as antileishmanials.

**Fig. 8.**
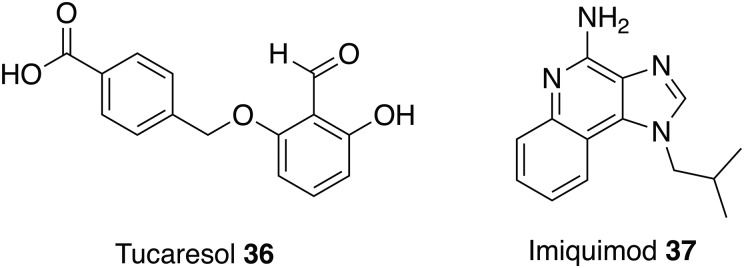
Antiviral drugs that could be repurposed as antileishmanials.

An antimicrobial is any compound that kills or inhibits the growth of a microorganism; this can include bacteria, fungi, viruses and protozoa. Antimicrobial drugs that were originally licensed for other indications have already been repurposed as antileishmanial therapies. For example, amphotericin B **5** and pentamidine **6** are antifungal medications and paromomycin **7** is used to treat the parasitic diseases, cryptosporidiosis and amoebiasis. These examples of successfully repurposed antimicrobials as treatments for leishmaniasis have encouraged further investigation into the leishmanicidal activity of other antimicrobial drugs (Figs 5–8, Table 1).

### Antifungals

As discussed above, the main use of amphotericin B **5** is in the treatment of a wide range of fungal infections but is now also used to treat VL. Liposomal amphotericin B **5** is the treatment of choice for immunocompetent patients in the Mediterranean region and the preferred drug for HIV/VL co-infection (Sundar and Chakravarty, 2010*a*). AmpB functions by binding to ergosterol disrupting the plasma membrane. This is the major sterol of the fungal plasma membranes and there are other antifungal azoles, which inhibit C14α-demethylase, an essential enzyme in the biosynthesis of ergosterol,. As the sterol biosynthesis pathway is conserved in *Leishmania* spp. parasites and is important for their survival (de Souza and Rodrigues, 2009), these antifungal azoles have the potential to be repurposed as antileishmanials. Fluconazole **19** and terbinafine **20**, two antifungal azoles, which have similar modes of actions, can be administered orally and possess low levels of toxicity; therefore, the use of these drugs as putative antileishmanials in clinical trials can be approached with some confidence. Oral fluconazole **19** was shown to be a safe and useful drug for the treatment of CL caused by *L. major* (Alrajhi *et al.* 2002) and was reported to be more efficacious in higher doses after a phase 2 clinical study (Emad *et al.* 2011). However, a recent phase 3 clinical trial of the use of high dose fluconazole **19** in the treatment CL caused by *L. braziliensis* and *L. guyanensis* was terminated due to a low cure rate, suggesting species dependent activity (ClinicalTrials.gov, 2015*a*). The potential for terbinafine **20** as a new treatment for CL was first described in 1997. Significantly, at the dosage used, no patients reported any side effects. However, the efficacy appears lower than other azole antifungals notably, ketoconazole **21** and itraconazole **22**, and further investigation is required to optimize the efficacy of the drug before it can be considered as an effective solution (Bahamdan *et al.* 1997). One method being investigated is using terbinafine **20** in combination therapies. For example, oral terbinafine **20** and cryotherapy has completed phase 1 of clinical trials for treatment of CL and results show a similar level of efficacy to glucantime **2** (Farajzadeh *et al.* 2015).

Positive results, coupled with attractive pharmacokinetic and safety profiles, with these antifungal azoles have encouraged the investigation of similar compounds. For example, itraconazole **22** and posaconazole **23** exhibit *in vitro* and *in vivo* activity against *L. amazonensis*, *L. donovani* and *L. infantum*. In addition, in a separate study, econazole **24** showed activity against *L. infantum chagasi* promastigotes and similar intramacrophage effectiveness to miltefosine **8** (Mesquita *et al.* 2014).

### Antiparasitics

A second source of antimicrobial compounds that have been exploited as antileishmanials are, not surprisingly, other antiparasitics. In one approach, a collection of 400 compounds obtained from Medicines for Malaria Venture (MMV) were screened for antileishmanial activity. Significantly, in contrast to many other such approaches, which use promastigote or axenic amastigotes, this study developed an assay using intracellular amastigotes, thus enabling screening against the most clinically relevant form of the parasite (Freitas-Junior *et al.* 2012). The results identified 14 antimalarial drugs that have antileishmanial activity. The compound of most interest was the anticancer drug, MMV666080 **25**, which, with an IC_50_ of 15 nm, is more potent and also less toxic than amphotericin B **5**. In addition, a series of substituted aminobenzimidazoles (**26–28)** were identified from this screen as active antileishmanials with IC_50_ values ranging from 61 to 134 nm. A current focus for these compounds is to validate the results using *in vivo* models (Khraiwesh *et al.* 2016).

A second set of studies has focused on the use of nitazoxanide **29**, an oral drug used in the treatment of infectious diarrhoea caused by the protozoan parasites, *Cryptosporidium parvum* and *Giardia lamblia*. The lethal action of nitazoxanide **29** against *L. infantum* involves upregulation of reactive oxygen species resulting in oxidative stress and parasite death (Mesquita *et al.* 2013). Nitazoxanide **29** also displays activity against *L. donovani* and *L.* amazonensis (Zhang *et al.* 2010; Nava-Zuazo *et al.* 2014), with spleen and liver parasite load being reduced by 86% in *L. donovani* infected mice (Zhang *et al.* 2010). In addition, nitazoxanide **29** demonstrates low cytotoxicity against mammalian VERO cell line and exhibits activity against *Giardia intestinalis* and *Trichomonas vaginalis*; therefore derivatives of nitazoxanide **29** would generally be attractive starting points for new antiparasitic drugs (Nava-Zuazo *et al.* 2014).

Due to the closer taxonomy lineage, it would be expected that anti-trypanosomal drugs would be more likely repurposed to leishmaniasis than other antiparasitics. However, apart from pentamidine used to treat African trypanosomiasis caused by *Trypanosoma gambiense*, no other anti-trypanosomal drugs including those used to treat Chagas Disease are presently used against leishmaniasis.

Whilst nitroaromatics, such as nitazoxanide **29**, are generally avoided in the pharmaceutical industry due to the known potential mutagenicity and carcinogenicity of the nitro group, recent research has revealed the potential of nitroaromatics to treat neglected parasitic diseases (Patterson and Wyllie, 2014). Reflecting this, another antiparasitic nitroaromatic compound, fexinidazole **30**, is being explored as a potential treatment for leishmaniasis. Fexinidazole **30** is in clinical trials for treating African trypanosomiasis (*T. brucei*) and Chagas disease (*T. cruzi*) where the mechanism of action is proposed to involve reductive activation of the nitro group by NADH-dependent bacterial-like nitroreductase. A study involving the overexpression of the leishmanial orthologue of the nitroreductase demonstrated that a similar mechanism occurs in *Leishmania* spp. Building on this fexinidazole **30** and its two predominant *in vivo* metabolites (fexinidazole sulfoxide and sulfone) displayed excellent activity against *L. donovani* promastigotes and axenic amastigotes. However, fexinidazole was inactive against intracellular amastigotes whereas fexinidazole sulfoxide and sulfone remained potent against intracellular amastigotes (both EC_50_ values equal to 5·3 *µ*m). When progressed into a murine model infected with *L. donovani* a 98·4% suppression of infection was observed with a single daily dose of fexindazole **30** for 5 days (Wyllie *et al.* 2012). With this positive data, fexinidazole **30** was progressed into clinical trials as a treatment for VL. In 2013, a phase II study tested fexinidazole **30** on 14 patients suffering with VL and all patients showed clinical improvement during treatment. Unfortunately, treatment relapses were observed and thus the study was terminated in 2014 due to lack of efficacy (ClinicalTrials.gov,^23,24^). However, fexinidazole **30** is now being investigated as a combination therapy with miltefosine **8** in Eastern Africa (DNDi, 2016).

Levamisole **31** is a heterocyclic compound that is immunoregulatory and an effective antihelminthic agent (Goldstein, 1978). Since the 1970s it has been studied as a therapy for CL (Butler, 1978) and a more recent study compared the effects of levamisole **31** and imidocarb **32**, veterinary medicine used to treat parasite infection, in murine models infected with *L. amazonensis*. The results from a number of parameters (IgG levels, vacuolar area, megakaryocyte count in spleen and parasite burden) demonstrate that imidocarb **32** has the most potential as a therapy for CL (Rodrigues *et al.* 2006).

### Antibacterials

The activity and safety profile of fexinidazole **30** sparked the search for other nitroimidazoles, which may have potential as oral treatments for VL. This identified the antituberculosis drugs PA-824 (**33–34)** and delamanid **35**, which can also undergo bioactivation by a nitroreductase, as potential leads (Patterson *et al.* 2013; Gupta *et al.* 2015; Patterson *et al.* 2016; Thompson *et al.* 2016). As with fexinidazole **30** the presence of the nitro group has been shown to be essential for antileishmanial activity (Patterson *et al.* 2013, 2016). However the nitroreductase found in *Mycobacterium tuberculosis* [deazaflavin (F420)-dependent nitroreductase (Ddn)] (Manjunatha *et al.* 2009), is absent in *Leishmania* and over-expression of the leishmanial nitroreductase did not provide enhanced sensitivity to the drugs, suggesting a different mode of action. This suggests the potential for future combination therapies involving fexinidazole **30** and these antituberculosis drugs. These differences are reinforced by the observation that whilst the *(S)*-enantiomer of PA-824 **33** is currently in phase II clinical trials for tuberculosis it is the *(R)*-enantiomer of PA-824 **34**, which shows superior activity against *L. donovani* parasites, both *in vitro* and *in vivo,* with a twice daily dose of *(R)*-PA-824 **34** at 100 mg kg^−1^ effectively curing a murine model of infection, suppressing infection by 99·9% (Patterson *et al.* 2013). More recently, the structurally similar compound delamanid **35** (R-enantiomer) has been demonstrated to display high activity *in vitro* against intracellular *L. donovani* amastigotes. In addition, a twice-daily oral dose of this nitroimidazole at 30 mg kg^−1^ for 5 days cured the mice infected with *L. donovani* (Patterson *et al.* 2016). Whilst these compounds appear to show considerable potential, a cautionary note arises from the history of the chimeric nitroimidazole, DNDI-VL-2098 **36**. This compound, following promising early results, was at the final stages of preclinical development for the treatment of VL. However, results from animal models showed a link between dose, length of treatment and testicular toxicity leading to a decision to halt progression (DNDi, 2015). Whether this is a compound specific effect or a class problem remains to be established.

### Antivirals

Tucaresol **36** is an investigational drug that has completed phase 2 clinical trials as a treatment for HIV (Gori *et al.* 2004; ClinicalTrials.gov, 2008) and, like the antiparasitic drug Levamisole **31**, is an immunomodulator that demonstrates antileishmanial activity *in vivo*. This novel immunomodulator was inactive in *L. donovani* intracellular amastigote assays but showed a reduction in parasite burden in mice infected with *L. donovani*. Tucaresol **36** could be delivered orally at an optimum dose of 5 mg kg^−1^ to produce a 44 to 62% suppression of liver amastigotes (Smith *et al.* 2000).

Another drug with immunomodulatory effects being studied as a therapy for leishmaniasis is imiquimod **37**. This immune-response modifier is a treatment for gentil warts caused by human papillomaviruses and works through activation of immune cells, including macrophages. Macrophages are the host cells of *Leishmania* and therefore in 1999 a study was conducted to determine if imiquimod **37** could be used as potential therapy for leishmania. *In vitro* results determined that imiquimod **37** could induce leishmanicidal properties in infected macrophages and a possible mode of action is through stimulated signal transduction increasing the synthesis of nitric oxide, which are toxic to the intracellular parasites. After the positive *in vitro* results it was important to test the capabilities of imiquimod **37** *in vivo*. *L. major* infected mice were treated with 5% imiquimod **37** cream and a significant reduction in the severity of the lesions was observed (Buates and Matlashewski, 1999) but the progression of this treatment to human trials demonstrated that topical application of imiquimod **37** alone was ineffective at treating CL (Seeberger *et al.* 2003). In 2005, a study focused on new approaches such as combination therapies and conducted a clinical trial of parental antimonial plus topical imiquimod **37** to treat patients with CL. Results from this study showed that 72% of patients treated with the combination containing **37** achieved a cure at 3 months *vs* 35% treated with the vehicle cream. In addition, accelerated healing and less prominent scarring was observed in the group treated with antimonial and imiquimod **37** compared with therapy with antimonial alone demonstrating that the next clinically available treatment for leishmaniasis might not rely on one but a combination of drugs (Miranda-Verástegui *et al.* 2005).

### Antihistamines

**Fig. 9.**
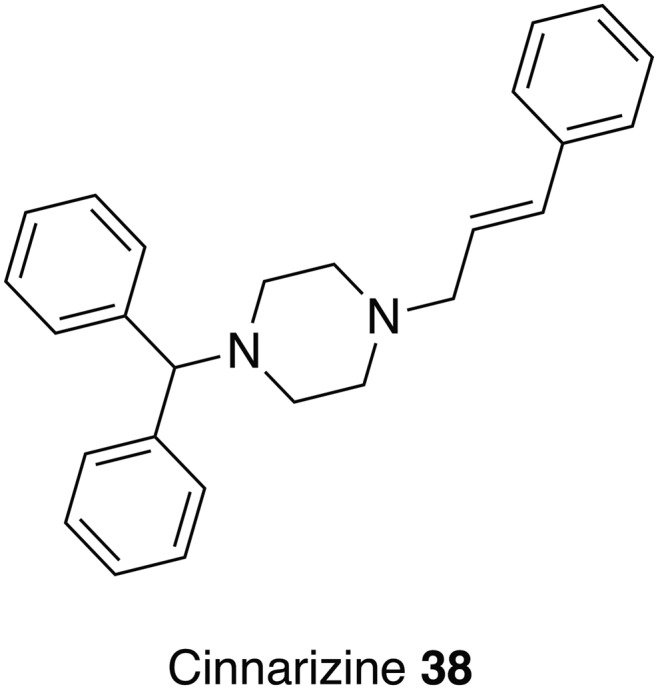
An antihistamine that could be repurposed as an antileishmanial.

Antiallergic compounds, such as quercetin found in *Kalanchoe* plant extracts, display potent antileishmanial effects. This reflects a capacity to restore the Th1/Th2T cell balance and also to inhibit mast cell histamine release (Cruz *et al.* 2008, 2012; Muzitano *et al.* 2008; Gomes *et al.* 2009; Mlcek *et al.* 2016). Consistent with these observations, a set of seven H1-antagonists were reported to display activity against *L. infantum* promastigotes with IC_50_ values in the range 13–84 *µ*m (Pinto *et al.* 2014). The activity recorded for cinnarizine **38** (Fig. 9, Table 1) was particularly noteworthy as, unlike the other compounds tested, it also demonstrated potency against intracellular amastigotes (EC_50_ = 21 *µ*m and NCTC cells EC_50_ = 87 *µ*m). Whilst, initial *in vivo* experiments using hamsters infected with *L. infantum* revealed a lack of efficacy, a liposomal formulation circumvented this problem and enabled effective treatment of infection in the liver but not the spleen, which could be ascribed to the higher parasite burden (amastigotes/gram of organ) in this latter organ. Cinnarizine **38** has also been described as a calcium channel blocker, which is significant because Reimão *et al.* (2010) demonstrated the activity of eight calcium channel blockers against intracellular *L. infantum* amastigotes (EC_50_ = 5–176 *µ*m) (Reimão *et al.* 2010). Further development of cinnarizine **38** will require the synthesis of more active analogues, an approach challenged by the fact that no target has yet been identified (Pinto *et al.* 2014).

In a separate study we have successfully developed an enzyme assay to explore the potential of the *Leishmania* sphingolipid synthase as a new antileishmanial drug target (Mina *et al.* 2010, 2011). This assay was employed to screen a set of 1040 pharmacologically active compounds selected from the National Institute of Neurological Disorders and Stroke (NINDS). Many of the most active and selective hits have reported antihistamine activity. As these maintained activity in infected cell models they have considerable potential for repurposing as target specific antileishmanials (Brown *et al*. in preparation).

### Central nervous system (CNS) active drugs

**Fig. 10.**
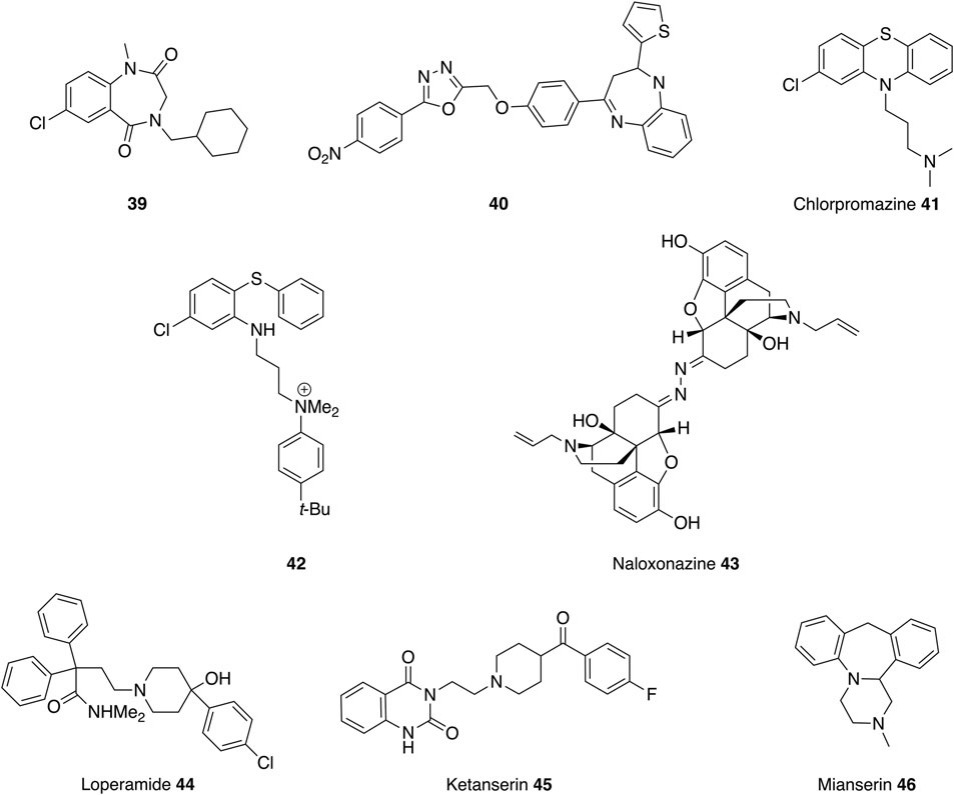
CNS drugs that could be repurposed as antileishmanials.

Benzodiazepines are sedatives used to treat anxiety, insomnia and seizures and various analogues have been reported to show activity against visceral and CL (Fig. 10). Compound **39,** structurally related to paullone, demonstrated efficacy against *L. donovani* amastigotes with no observed toxicity (Clark *et al.* 2007). In a separate study, benzodiazepine **40** was effective against *L. mexicana* promastigotes with a better IC_50_ value than that observed for miltefosine **8** (Navin *et al.* 2017). However, metabolic studies using rat hepatocytes and microsomal preparation showed that the potential of these compounds is limited by extensive and rapid metabolism. Therefore, further rounds of medicinal chemistry optimisation will be required (Thi *et al.* 2009).

Tricycylic heterocycles are part of numerous therapeutic agents and therefore important structures in medicinal chemistry (Marcu *et al.* 2016). Chlorpromazine **41** is perhaps the best known of the phenothiazine drugs (tricycylic heterocycles) for treatment of neurological disorders. In 1984 this compound was identified as having activity against *L. donovani in vitro* and *in vivo* (Pearson *et al.* 1984) and in a separate study also showed it could kill *L. mexicana*, *L. aethiopica* and *L.major* promastigotes at a concentration of 7·5 *µ*g mL^−1^ (El-On *et al.* 1986). The same study determined the *in vivo* effect for chlorpromazine **41** in a murine model infected with *L. major* and *L. mexicana* and demonstrated leishmanicidal effect in the spleen but not in the skin lesion (El-On *et al.* 1986). Therefore, like pentamidine, this compound was shown to be effective against VL but with only partial effect against the cutaneous form. Chlorpromazine **41** will probably not be useful in the treatment of VL due to toxicity concerns but these results have led to the investigation of structurally similar compounds (Chan *et al.* 1998; Mukherjee *et al.* 2012; Marcu *et al.* 2016) to explore the SAR. For example, chlorpromazine analogues with a quaternary nitrogen atom **42** have demonstrated antileishmanial, antitrypanosomal and antimalarial activity (Khan *et al.* 2000; Parveen *et al.* 2005). These analogues are trypanothione reductase inhibitors, which are likely to affect the redox defense of the parasite and hence increase the parasites sensitivity to redox-damaged-based drugs. In addition, the enzyme, trypanothione reductase, is present in *Leishmania* and *Trypanosoma* but not the mammalian hosts providing the opportunity to design a selective inhibitor (Khan *et al.* 2000; Parveen *et al.* 2005).

HTS against the *L. major* inositol phosphorylceramide synthase (IPCS) formatted into a cell-based yeast assay identified a class of benzazepanes as potent inhibitors of the enzyme with good activity against both *L. major* and intramacrophage *L. donovani* with good levels of selectivity *vs* Human THP-1 cells; on target effects were shown by comparison with a mutant *L. major* strain with redundant sphingolipid synthase activity. However, again further progression requires improvements to the pharmacokinetic profile of these compounds (Norcliffe *et al.*, in prep).

The opioid receptor antagonist naloxonazine **43** was identified in a screen comparing promastigote and amastigote activity in which it was uniquely the only compound identified that showed activity against the intracellular amastigote and not the promastigote. Curiously, the structurally related opioid naloxone (Narcan^®^) was completely inactive against both parasitic forms. Since homologs of opioid receptors have not been identified in the Leishmania genome it is possible that opioids are involved in modulation of host resistance to parasite infection. In support of this, Loperamide **44**, a *μ*-opioid receptor agonist, was also identified in this study as inhibiting parasite growth with greater activity against the intracellular stage of the parasite (De Muylder *et al.* 2011).

Finally, antileishmanial activity was observed when the broad spectrum antidepressants, ketanserin **45** and mianserin **46**, were screened against *L. donovani* promastigotes and intramacrophage amastigotes. Amongst other enzymes these compounds are known to target 3-hydroxy-3-methylglutaryl coenzyme A reductase (HMGR). This is the rate-limiting enzyme of the sterol biosynthesis, which is also the putative pathway targeted by amphotericin B **5**. In support of this proposal both compounds inhibited recombinant *L. donovani* HMGR, although given the diverse reported activities, other modes of action cannot be discounted (Dinesh *et al.* 2014; Singh *et al.* 2014*b*).

### Other drugs

Similar to ketanserin **45** and mianserin **46** discussed above, statins target HMGR. Statins are currently on the market to reduce cardiovascular disease by lowering the level of low-density lipoprotein cholesterol (‘bad cholesterol’) in the blood. Studies have shown that statins also have the potential to be repurposed as antileishmanials (Kumar *et al.* 2016; Parihar *et al.* 2016). For example, mevastatin **47** also inhibits recombinant *L. donovani* HMGR enzyme with an IC_50_ value of 42·2 ± 3·0 *µ*m. It also shows correspondingly good activity against both *L. donovani* promastigotes and intracellular amastigotes with no toxicity exhibited towards the host cell line (Dinesh *et al.* 2015).

**Fig. 11.**
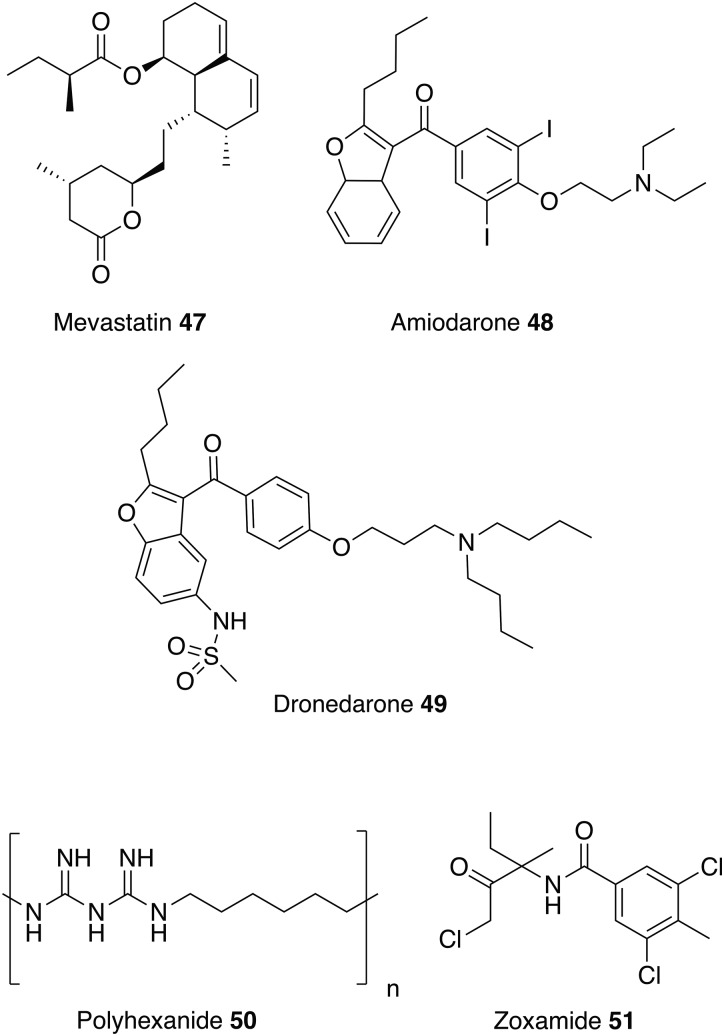
Other drugs that could be repurposed as antileishmanials.

Amiodarone **48** and dronedarone **49**, (Fig. 11) used to treat cardiac arrhythmias, have antileishmanial activity against *L. mexicana* promastigotes and intramacrophage amastigotes, and inhibit oxidosqualene cyclase (OSC), another enzyme essential for ergosterol biosynthesis. However, as with the antihistamines discussed above, these drugs are also known to disrupt Ca^2+^ homeostasis in *Saccharomyces cerevisiae* and *Trypanosoma cruzi,* so may have multiple modes of action (Serrano-Martin *et al.* 2009*a*). The *in vivo* activity of amiodarone **48** was investigated in a murine model infected with *L. mexicana*. amiodarone **48** was given as an oral therapy at 50 mg kg^−1^ day^−1^ and although preventing the development of lesions during the course of treatment, reoccurrences of the infection were observed once treatment had ended. Amiodarone **48** was then tested in combination with miltefosine **8** at a dose of 50 and 20 mg kg^−1^ day^−1^ respectively, and showed permanent control of lesion size. Hence, using this drug in combination with currently available antiparasitics could lower the dosage and reduce the known side effects of amiodarone (e.g. cardiotoxicity, thyroid dysfunction and pulmonary fibrosis) (Serrano-Martín *et al.* ^106,107^). Alternatively, dronedarone **49** appears to have great potential to be repurposed as it has greater antiparasitic activity coupled with lower mammalian toxicity than amiodarone **48** (Benaim *et al.* 2014).

This review has focused on repurposing synthetic small molecules, though there are studies that have investigated polymers and natural products. For instance, a recent study confirmed that polyhexanide **50**, a wound antiseptic and disinfectant, has antileishmanial activity with IC_50_ values of 0·41 *µ*m against *L. major* promastigotes, 69-fold more potent than miltefosine **8**, and 4 *µ*m against intracellular *L. major* amastigotes. Polyhexanide **50** is thought to kill the parasite by disruption to membrane structure and selective chromosome condensation and damage. Interestingly, it was also discovered that this cationic polymer can be used as a vehicle to transport cargoes to the macrophage and thus has the ability to deliver immunomodulatory agents (Firdessa *et al.* 2015).

Whilst the focus of this review has been on repurposing human therapeutics, there is the potential for investigating other groups of bioactive chemicals, including both agrochemicals and veterinary medicines for human use as treatments for neglected diseases. For example, more than 600 commercial agrochemicals were screened against parasitic protozoans and zoxamide **51** was found to be the most effective against *L. donovani* promastigotes. Zoxamide **51** is an oomyceticidal compound used in fruit and vegetables, and acts through the inhibition of microtubule formation. Microtubule inhibitors may be a class of potential antileishmanials since tubulin is one of the most abundant leishmanial proteins (Kapoor *et al.* 1999). Zoxamide **51** killed the promastigotes with an IC_50_ value of 250 nm (Witschel *et al.* 2012). Although explored in other contexts (Andrews *et al.* 2014), there has been little reported effort to repurpose veterinary medicines for leishmanaisis, possibly due to the potential human toxicity issues which could arise and negate the benefits of the repurposing approach.

### Concluding remarks

The repositioning of old drugs for new uses is not a new concept and is a particularly attractive approach for neglected tropical diseases. Here the use of approved drugs is particularly beneficial as clinical trials have already successfully been conducted and often their patents have expired leading to shorter, cheaper discovery pipelines. As discussed in the various sections above, a wide range of structures have been explored for antileishmanial activity. Significantly, most of the medicines used to treat leishmaniasis, both on the market or under development in the drug discovery process, were initially intended for other indications. Furthermore, more novel approaches show drug repositioning is extending beyond the scope of human medication as investigation into the human use of agrochemicals and veterinary therapeutics is underway. However, drug repurposing is limited by the number of approved drugs and compounds with established pharmacokinetic data, in addition the repositioning discovery process tends to mean that there is less chance of finding treatments that act through a new mechanism of action. Overall, whilst not the sole solution, drug repositioning represents a valuable, relatively fast and cost-effective, strategy for developing essential new therapies, particularly for neglected tropical diseases such as leishmaniasis.
